# Urolithin A Inhibits the Catabolic Effect of TNFα on Nucleus Pulposus Cell and Alleviates Intervertebral Disc Degeneration *in vivo*

**DOI:** 10.3389/fphar.2018.01043

**Published:** 2018-09-18

**Authors:** Huiyong Liu, Honglei Kang, Chao Song, Zuowei Lei, Li Li, Jianfeng Guo, Yong Xu, Hanfeng Guan, Zhong Fang, Feng Li

**Affiliations:** ^1^Department of Orthopaedic Surgery, Tongji Hospital, Tongji Medical College, Huazhong University of Science and Technology, Wuhan, China; ^2^Department of Radiology, Tongji Hospital, Tongji Medical College, Huazhong University of Science and Technology, Wuhan, China

**Keywords:** urolithin A, intervertebral disc degeneration, nucleus pulposus cells, senescence, TNFα

## Abstract

Low back pain (LBP) is a common worldwide disease that causes an enormous social economic burden. Intervertebral disc degeneration (IDD) is considered as a major cause of LBP. The process of IDD is complicated and involves both inflammation and senescence. The production of pro-inflammatory cytokines, including tumor necrosis factor (TNF)α and interleukin (IL)-1β, is increased in the degenerating intervertebral disc, inducing extracellular matrix degradation. Urolithin A (UA) is a metabolite compound resulting from the degradation of ellagitannins by gut bacteria. UA has been reported to be useful for the treatment of diseases associated with inflammation, senescence, and oxidative damage. Therefore, we hypothesized that UA may be an effective treatment for IDD. This study examined the effects of UA on IDD *in vitro* and *in vivo* and explored their underlying mechanisms. Our findings indicated that UA could attenuate cellular senescence induced by hydrogen peroxide in nucleus pulposus cells. UA treatment decreased TNFα-induced matrix metalloproteinase production and the loss of collagen II. At the molecular level, UA considerably blocked the phosphorylation of the extracellular signal-regulated kinase, c-JUN N-terminal kinase, and Akt pathways. *In vivo* study illustrated that UA treatment could ameliorate IDD in a needle-punctured rat tail model, which was evaluated by X-ray imaging, magnetic resonance imaging, and histological analysis. Thus, the results of our study revealed that UA may be a useful therapeutic agent for the treatment of IDD.

## Introduction

Low back pain (LBP) is the most frequent diseases leading to a low quality of life (Bressler et al., 1999; Hoy et al., 2012; Prince et al., 2015). It causes suffering and distress to patients and brings an enormous economic burden on society. Many diseases can cause LBP, such as degenerative disc disease, spinal stenosis, spondyloarthritis, and muscle strain, among which intervertebral disc degeneration (IDD) is the major contributor of LBP. The intervertebral disc is a cartilaginous structure that comprises the outer annulus fibrosis (AF), inner nucleus pulposus (NP), and cartilage endplates. The AF and NP provide the properties of flexibility to withstand mechanical loading. The AF is composed by a series of concentric rings or lamellae that are made up of highly collagen fibers. The gelatinous NP is the major functional composition of the intervertebral disc. It consists of chondrocyte-like NP cells that produce abundant extracellular matrix (ECM) such as collagen I, collagen II, and proteoglycan (Wei et al., 2014). The disc homeostasis depends on the balance and interactions of cells, ECM, biomechanical stress, and others. IDD is a multifactorial process characterized by serial progressive changes in the morphology, biochemistry, components, and biomechanical function of intervertebral discs (Urban and Roberts, 2003). Although the mechanism of IDD is not fully understood, altered mechanical loading, degeneration of the ECM, increased secretion of inflammatory factors, excessive senescence, and apoptosis of NP cells have been proven to play an important role in its development (Stokes and Iatridis, 2004; Le Maitre et al., 2007; Kepler et al., 2013).

Ellagitannins (ETs) constitute a diverse class of hydrolyzable tannins present mainly in some fruits, such as pomegranates, blackberries, and strawberries (Bakkalbasi et al., 2009). They are hydrolyzed in the gut to release ellagic acid (EA). Urolithins, which are thought to be the intestinal microbial metabolites of both ETs and EA, include urolithin A (UA), urolithin B (UB), urolithin C (UC), and urolithin D (UD). Recently, a few studies have documented the biological effects of urolithins *in vitro* and *in vivo*, including antiproliferation in cancer, anti-inflammation, anti-oxidant activity, benefits on lipid metabolism, and prolonged lifespan (Bialonska et al., 2009; Ishimoto et al., 2011; Gimenez-Bastida et al., 2012; Ryu et al., 2016; Zhang et al., 2016). UA has been reported to exert *in vitro* and *in vivo* anti-inflammatory actions in the colon through inhibit NF-κB pathway and MAPK pathway activation (Gonzalez-Sarrias et al., 2010) and ameliorate TNFα-induced inflammation in human aortic endothelial cells (Gimenez-Bastida et al., 2012). The increased inflammatory cytokines and senescence are involved in the process of IDD through NF-κB pathway, MAPK pathway, and PI3K/Akt pathway. Nevertheless, the effect of urolithins on IDD has not been investigated to date. Here, the present study was designed to investigate whether UA can alleviate IDD and elucidate the molecular mechanisms involved in this progress using both *in vitro* and *in vivo* models.

## Materials and Methods

### Reagents

Urolithin A was obtained from Santa Cruz Biotechnology (Santa Cruz, CA, United States) and was diluted in dimethylsulfoxide (DMSO) and stored at -20°C. Recombinant rat tumor necrosis factor (TNF)α was purchased from R&D Systems (Minneapolis, MN, United States). H_2_O_2_ and collagenase type II were purchased from Sigma–Aldrich (St. Louis, MO, United States).

### Animals

For the IDD rat model, 12-weeks-old male Sprague–Dawley (SD) rats were obtained from the Experimental Animal Center of Tongji Medical College (Wuhan, China). All the animal studies were authorized by the Ethics Committee on Animal Experimentation of Tongji Medical College (No. TJ-A20161204).

### Isolation and Culture of Nucleus Pulposus (NP) Cells

Primary rat NP cells were isolated and cultured using a method described previously (Li et al., 2016). Briefly, 12-weeks-old male SD rats were euthanized by injection of an excess amount of sodium pentobarbital. The gel-like NP tissues were carefully separated from the lumbar discs and digested with 0.01% collagenase type II for 4–6 h at 37°C. The isolated cells were cultured in DMEM/F12 (Invitrogen, Carlsbad, CA, United States) containing 10% fetal bovine serum (FBS), 100 U/ml penicillin, and 100 μg/ml streptomycin. The second- or third-passage NP cells were used in all the *in vitro* experiments. The medium was changed every 2 days.

### Cell Viability Assay

NP cells were seeded in 96-well plates at a density of 1 × 10^4^ cells per well and were incubated in complete medium overnight. Cells were then treated as indicated. The cytotoxic assay was performed using Cell Counting Kit-8 (CCK8; Beyotime, Jiangsu, China) according to standard manufacturer’s protocol after 1, 3, and 5 days.

### Quantitative Real-Time Reverse Transcription-Polymerase Chain Reaction (qRT-PCR)

qRT-PCR was performed as described previously (Guan et al., 2010, 2013). Briefly, total RNA was extracted from NP cells using TRIzol reagent (Invitrogen, Carlsbad, CA, United States). Next, 1 μg of total RNA was used to synthesize cDNA using the 5X All-In-One RT MasterMix (ABM, Vancouver, BC, Canada). The templates were amplified using the SYBR Green PCR Master Mix (Kapa Biosystems, Wilmington, MA, United States) in the CFX96 Touch^TM^ Real Time PCR system (Bio-Rad, Hercules, CA, United States) according to the manufacturers’ instructions. The relative expression of a target gene was normalized to the standard reference gene glyceraldehyde 3-phosphate dehydrogenase (GAPDH). The primer sequences are listed in **Table 1**.

**Table 1:** Sequences of primers used in the qPCR.

Name	Sequence (5^′^–3^′^)
GAPDH (rat)	Forward GGCACAGTCAAGGCTGAGAATG
	Reverse GGTGGTGAAGACGCCAGTA
Collagen II (rat)	Forward CGAGGCAGACAGTACCTTGA
	Reverse TGCTCTCGATCTGGTTGTTC
Aggrecan (rat)	Forward CTTCCCAACTATCCAGCCAT
	Reverse TCACACCGATAGATCCCAGA
MMP3 (rat)	Forward GCTCATCCTACCCATTGCAT
	Reverse GCTTCCCTGTCATCTTCAGC
MMP13 (rat)	Forward GTGTGACAGGAGCTAAGGCA
	Reverse ATGAACATGGAGGAGCATGA

*GAPDH, glyceraldehyde 3-phosphate dehydrogenase; MMP3, matrixmetallopeptidase-3; MMP13, matrixmetallopeptidase-13.*

### Western Blot Analysis

Total protein was isolated from cultured NP cells using RIPA Lysis buffer (Boster, Wuhan, China) with 100 μM phenylmethylsulfonyl fluoride, and the protein concentration was measured using the bicinchoninic acid assay. Immunoblotting was performed as described previously (Guan et al., 2010, 2013). The primary antibodies against p65, phosph-p65, extracellular signal-regulated kinase (ERK), phospho-ERK, c-JUN N-terminal kinase (JNK), phosph-JNK, p38, phosph-p38, Akt, and phosph-Akt were obtained from Cell Signaling Technology (Danvers, MA, United States). The monoclonal antibodies against collagen II, matrix metalloproteinase (MMP)3, MMP13, and GAPDH were purchased from Abcam (Cambridge, MA, United States). The secondary antibodies were purchased from Jackson Immuno Research (West Grove, PA, United States). Signals were visualized using enhanced chemiluminescence according to the manufacturer’s recommendations, and the band intensities were measured using ImageJ software (National Institutes of Health, Bethesda, MD, United States).

### SA-β-Gal Staining

The SA-β-gal activity of the cultured NP cells were measured using a senescence β-galactosidase staining kit (Beyotime, China). Briefly, NP cells were seeded in a six-well plate (1 × 10^5^ cells/well) and were pretreated with UA for 2 h and then were treated with H_2_O_2_ (50 μM) for 2 h. SA-β-gal staining was performed according to the manufacturer’s instructions. The cells were then observed under an Olympus BX51 microscope and were analyzed using ImageJ software.

### Surgical Procedure

Thirty rats were divided randomly into three equal groups (*n* = 10 per group): sham-operated mice (control group), punctured and DMSO-treated mice (IDD group), and punctured and UA-treated mice (UA group). As described previously (Li et al., 2016), SD rats were euthanized by injection of sodium pentobarbital, and needles (21-gage) were used to puncture the coccygeal intervertebral discs (Co7-8, Co8-9) percutaneously at a depth of 5 mm, followed by rotation at 360° and holding for 30 s. One day after surgery, the rats were given foods containing UA for 4 weeks. UA was mixed with the rodent diet at a concentration of 0.25 g per kg of diet, corresponding to a dose of 25 mg/kg/day (Ryu et al., 2016).

### Radiographic Study of the Disc Height and Magnetic Resonance Imaging (MRI)

Radiographic and magnetic resonance imaging (MRI) scans of the tails were acquired before puncture and 4 weeks after puncture. After inducing anesthesia, each rat was placed in the prone position on a GE X-ray system (GE Mammography DMR Bucky 18 x 24; GE Healthcare, Little Chalfont, United Kingdom) with their tails straight. X-ray scans were performed at a collimator-to-film distance of 66 cm, an exposure of 63 mA, and a penetration power of 65 kV. The disc height was measured using the ImageJ software and disc height index (DHI) was calculated as previously described (Han et al., 2008; Keorochana et al., 2010; Mao et al., 2011).

Magnetic resonance imaging scans were performed to evaluate the structural and signal change in T2-weight images using a 3.0-T clinical MR scanning system (GE Discovery MR 750; GE Healthcare). The T2 sagittal sections were obtained using the fast-spin echo sequence with the following settings: a repetition time (TR) of 2000 ms, an echo time (TE) of 36 ms, a matrix of 256 × 256, a field of view (FOV) of 8.0 cm × 1.0 cm, a slice thickness of 1 mm, and an interslice gap of 1 mm. The MRIs were evaluated by blind observers using the classification method reported by Pfirrmann et al. (2001).

### Histopathologic Analysis

After 4 weeks of puncture, all the rats were euthanized with an excess amount of 6% chloral hydrate and the tails were collected. The spines were fixed in 4% paraformaldehyde, decalcified in 10% EDTA for about 6 weeks, dehydrated, and embedded in paraffin. The tissues were cut into 5-μm sections. The slices were stained with HE, Alcian blue, and safranin-O-Fast Green stains. Histological images were examined by blind observers under the microscope and were evaluated as described previously (Mao et al., 2011).

### Statistical Analysis

All the data were expressed as means ± standard deviation (SD) from three independent experiments. Student’s *t*-test was used for the comparisons between two groups. One-way analysis of variance (ANOVA) was used for comparisons involving more than two groups. All statistical analyses were carried out using SPSS 17.0 software (SPSS, Inc., Chicago, IL, United States). *p* < 0.05 was considered statistically significant.

## Results

### Effect of UA on the Proliferation and Senescence of NP Cells

**FIGURE 1:**
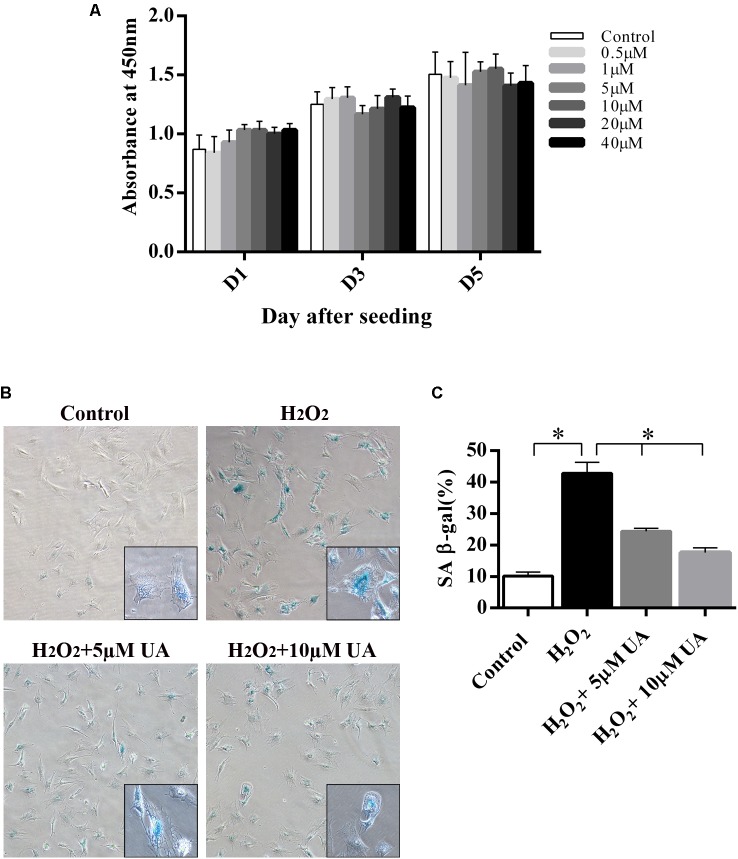
The effect of UA on NP cell viability and UA treatment inhibits H_2_O_2_-induced cell senescence. **(A)** 1 × 10^3^ NP cells were seeded in 96-well plates and treated with UA at the indicated concentrations for 5 days. Cell viability was determined by the CCK8 kit. **(B,C)** Results of the SA-β-gal staining assay (original magnification: ×100). NP cells were pretreated with different concentrations of UA for 12 h and then were treated with 50 μM H_2_O_2_ for 2 h. SA-β-gal staining was performed. The data represent the means ± SD. Significant differences between the treatment and control groups are indicated as ^∗^*P* < 0.05 compared with the control group.

We first tested the potential toxicity of UA on cultured NP cells using the CCK8 assay. As shown in **Figure 1A**, UA did not significantly affect the proliferation of NP cells that treated with UA at ≤ 40 μM for 5 days. For all subsequent *in vitro* experiments, we decided to treat NP cells with 5, 10, and 20 μM UA as indicated. During the IDD, the senescent and apoptosis NP cells are increased (Ding et al., 2013). It was reported that oxidative stress could induce cell senescence, including that of NP cells (Chen et al., 2007; Dimozi et al., 2015). Next, we tested whether UA could protect NP cells from hydrogen peroxide (H_2_O_2_)-induced senescence using SA-β-gal staining. As shown in **Figures 1B,C**, significantly increased SA-β-gal-positive senescent NP cells were observed following H_2_O_2_ treatment, whereas UA could reverse this change.

### UA Attenuates TNFα-Induced NP ECM Degradation

**FIGURE 2:**
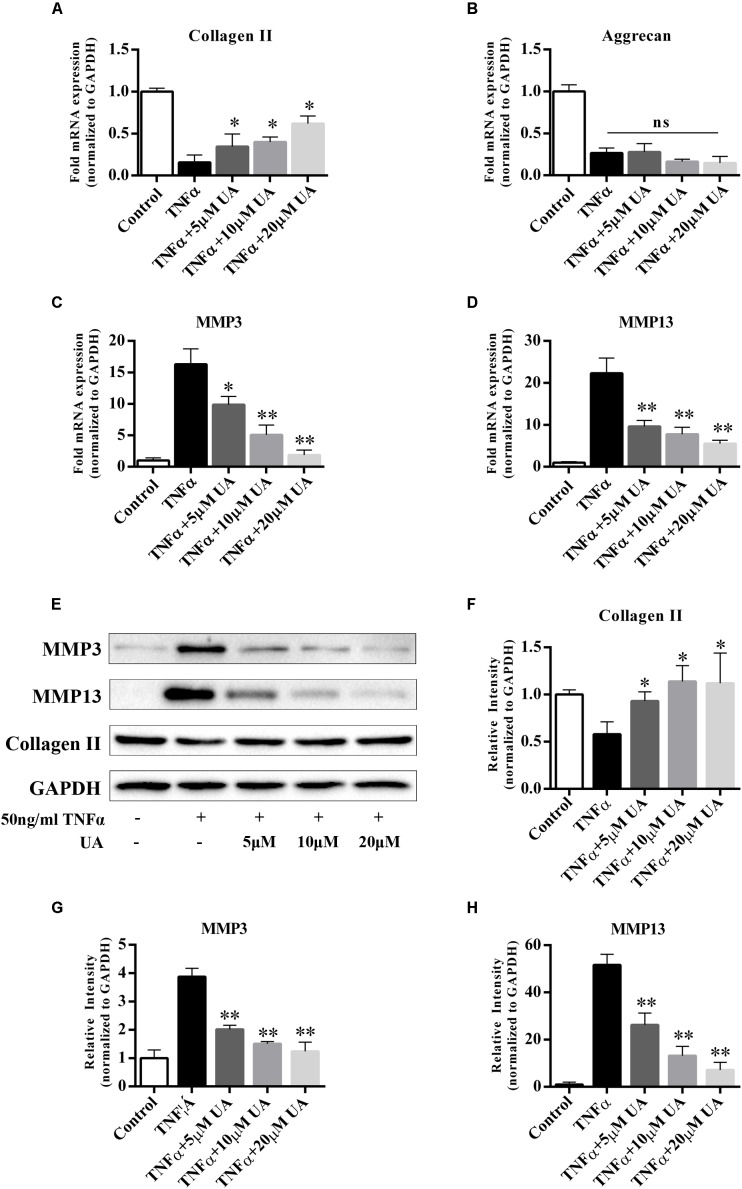
Effects of UA on anabolic/catabolic genes and protein expression in NP cells induced by TNFα. NP cells were pretreated with UA at the indicated concentrations for 2 h, and then 50 ng/ml of TNFα was added in serum-free media for 48 h. **(A–D)** The mRNA expression levels of collagen II, aggrecan, MMP3, and MMP13 were measured by qPCR. **(E–H)** The protein expression was measured by using western blotting. The results were quantified using ImageJ software. Gene expression was normalized by GAPDH expression. The data represent the means ± SD. ^∗^*P* < 0.05, ^∗∗^*P* < 0.01 compared with samples only treated with TNFα.

It was reported that UA has anti-inflammatory properties. The degenerated intervertebral disc is characterized by an increase in the levels of inflammatory cytokines secreted by disc cells, especially TNFα and IL-1β (Risbud and Shapiro, 2014). They induce the expression of MMPs, especially MMP3 and MMP13, which promote ECM degradation (Roberts et al., 2000; Seguin et al., 2005). To investigate whether UA affects TNFα-induced ECM degradation, we analyzed the expression of catabolic and anabolic factors after treatment of the NP cells with UA and TNFα for 48 h. The mRNA and protein expression of the ECM synthesis genes (collagen II and aggrecan) and ECM degradation genes (MMP3 and MMP13) of NP cells were tested by using qRT-PCR and western blotting. As shown in **Figures 2A–D**, TNFα treatment significantly reduced the mRNA expression of collagen II and aggrecan but strongly upregulated the expression of MMP3 and MMP13. UA treatment increased the mRNA expression of TNFα-induced inhibition of collagen II and decreased the expression of MMP3 and MMP13, but the mRNA expression of aggrecan was not reversed (**Figure 2B**). The western blotting results also confirmed that UA upregulated the expression of collagen II and strongly attenuated the expression of MMP3 and MMP13 (**Figures 2E–H**).

### UA Inhibits TNFα-Induced Mitogen-Activated Protein Kinase (MAPK)s and PI3K/Akt Pathway Activation

**FIGURE 3:**
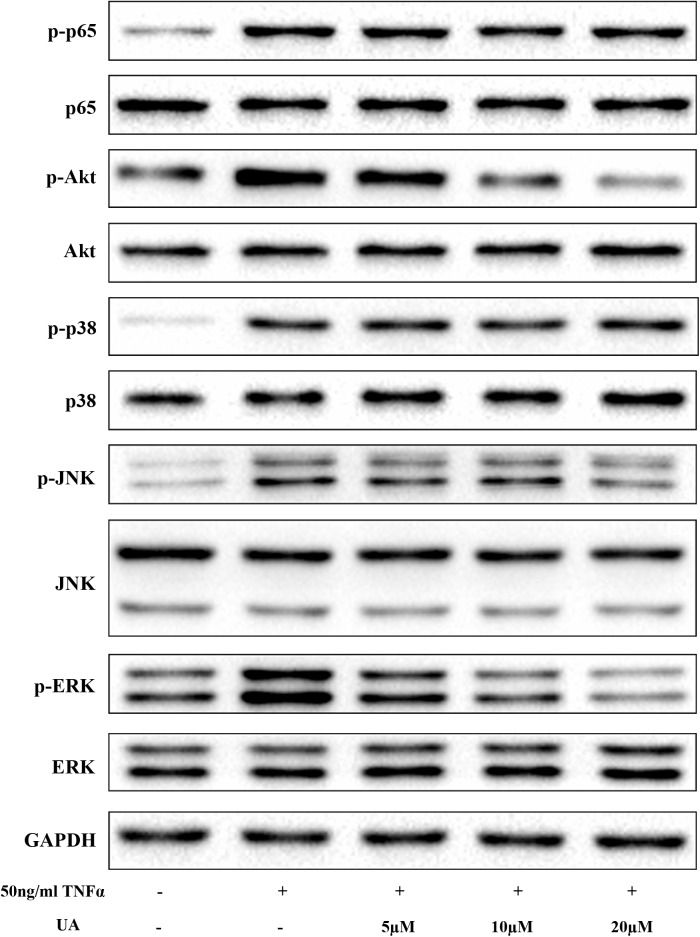
The suppressive effect of UA on the TNFα-induced MAPK and PI3K/Akt pathways. The NP cells were pretreated with the indicated concentration of UA for 2 h. Subsequently, 50 ng/ml of TNFα was added, and the cells were incubated for 30 min. The total protein was then extracted.

Activation of the nuclear factor (NF)-κB, MAPK, and the PI3K/Akt pathways play pivotal roles in IDD (Wuertz et al., 2012). To examine the effects of UA on the TNFα-induced signaling pathway, we tested the major molecular pathways after 1 h of 50 ng/ml of TNFα and different concentrations of UA stimulation (**Figure 3**). As expected, the MAPK, PI3K/Akt, and NF-κB pathways were strongly activated after TNFα treatment, as indicated by upregulated the phosphorylation level of their core proteins. The western blot analysis showed that UA could suppress the phosphorylation of ERK, JNK and Akt. However, the NF-κB p65 and p38 MAPK pathways were not significantly influenced by UA in TNFα-induced NP cells.

### UA Ameliorates Intervertebral Disc Degeneration *in vivo*

**FIGURE 4:**
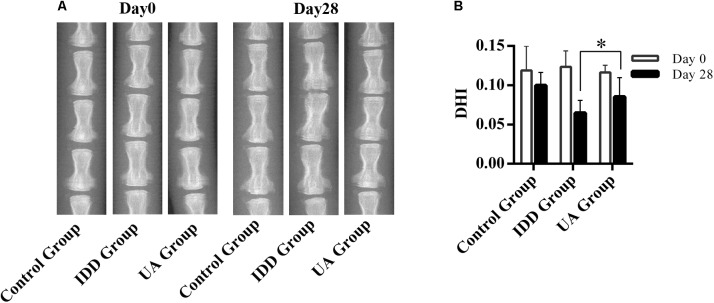
UA treatment alleviates puncture-induced disc space narrowing. **(A,B)** X-ray images of rat tails were acquired at days 0 and 28 after disc puncture. The data represent the means ± SD. ^∗^*P* < 0.05 compared with the IDD group.

**FIGURE 5:**
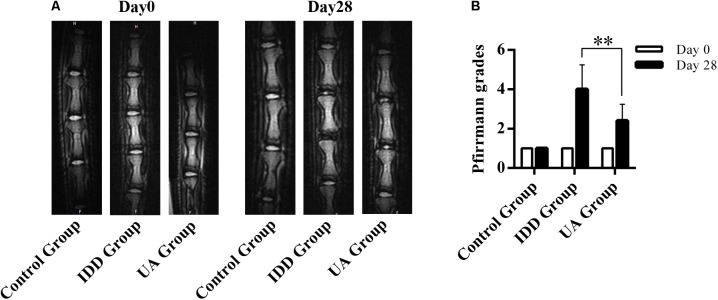
UA treatment improves the MRI grading of IDD. **(A,B)** T2-weighted image of rat tails. Grading of disc degeneration was evaluated according to a grading system reported by Pfirrmann et al. The data represent the means ± SD. ^∗∗^*P* < 0.01 compared with the IDD group.

The needle-punctured model was used to mimic the degenerative process *in vivo* (Li et al., 2016). After the procedure, rats were given UA or DMSO for 4 weeks through their food. X-ray images and MRI were acquired at 4 weeks after the surgery and were compared with images acquired before the procedure. 4 weeks later, the X-ray images of the IDD group (puncture + DMSO treatment) indicated a loss of disc height and osteophyte formation compared with those of the control group (only DMSO treatment). By contrast, the UA group (puncture + UA treatment) showed no significant disc space narrowing (**Figure 4**). The DHI decreased from 0.123 ± 0.021 to 0.065 ± 0.016 in the IDD group after puncture. However, there was a slight decline in the UA group (from 0.116 ± 0.009 to 0.086 ± 0.025). Additionally, the levels of disc degeneration were also assessed by MRI according to the MRI grade scoring system reported by Pfirrmann et al. (2001). At 4 weeks after puncture, the T2-weighted signal intensity was markedly higher and the distinction between the nucleus and annulus was clearer in the UA group than in the IDD group (**Figure 5**). In addition, the Pfirrmann grade scores were lower in the UA treatment group than in the IDD group.

**FIGURE 6:**
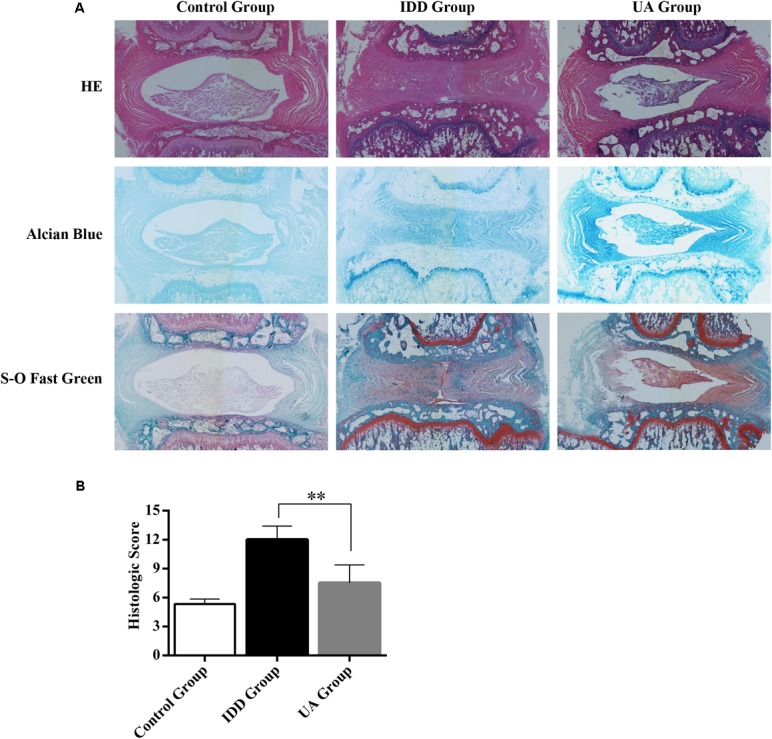
Representative histological sections of the intervertebral disc. **(A)** HE staining, Alcian Blue staining, and Safranin O-Fast Green staining were performed 4 weeks after surgery. The stained images were magnified 40×. *N* = 10 per group. **(B)** Histologic score of the disc in each group. The data represent the means ± SD. ^∗∗^*P* < 0.01 compared with the IDD group.

As shown in **Figure 6A**, hematoxylin and eosin (HE) staining demonstrated abundant gel-like NP and an intact, well-organized AF in the control group. The inner NP displayed a mix of stellar-shaped cells with a proteoglycan matrix located at the periphery. The border between the NP and AF was clear. However, clear histological changes were observed in the IDD group after puncture. The NP constituted less than 25% of the disc area, and it was irregular. The disc was occupied by fibrocartilaginous, disorganized tissue. The histologic score of the IDD group was higher than that of the control group. UA treatment markedly alleviated disc destruction compared with that in the IDD group (**Figure 6B**). Alcian blue staining showed deep blue in the NP and inner layers of the AF in the UA group, indicating pronounced expression of proteoglycan and collagen in the UA group compared with that in the IDD group. Our data clearly shown that the histological grade of the UA group was better than that of the IDD group.

## Discussion

The intervertebral disc is the largest avascular tissue and the nutrients were supplied by infiltration of the surrounding tissues. IDD leads to a series of changes in tissue physiology, morphology, and biomechanics, including ECM remolding, loss of collagen and aggrecan, increased cell senescence and death, and decreased water content and disc height (Antoniou et al., 1996; Roughley, 2004; Roberts et al., 2006). IDD is the main factor contributing to chronic LBP. The treatment of IDD associated with LBP remains controversial because the detailed mechanism of IDD is not fully understood and current treatment is not ideal. Novel therapeutic strategies must be explored to alleviate pain and limit further IDD. Previous studies have shown that the inhibition of inflammatory factors and oxidative stress, including cyclooxygenase 2 (COX2; Van Dijk et al., 2015; Willems et al., 2015), TNFα (Seguin et al., 2005; Li et al., 2016), interleukin (IL)-1β (Daniels et al., 2016), and reactive oxygen species (ROS; Suzuki et al., 2015) could relieve pain and prevent IDD. A few studies have shown that UA has potent of anti-inflammatory, anti-oxidative, anti-cancer, and lifespan-prolonging activities, which inhibit multiple pathways, including ROS, lipopolysaccharide (LPS), and inducible nitric oxide synthase pathways (Bialonska et al., 2009; Ryu et al., 2016; Spigoni et al., 2016; Guada et al., 2017; Boakye et al., 2018; Xu et al., 2018). In this context, we show that UA exhibits pharmacological anti-ECM degradation and anti-senescence effects *in vitro* and *in vivo* through inhibit the activation of MAPK signaling pathway and PI3K/Akt signaling pathway. Administering UA may be a valid therapeutic strategy.

Previous studies have implicated the pro-inflammatory cytokine TNFα as a critical catabolic factor in IDD because TNFα has been detected in degenerate intervertebral disc, and it modulates matrix production (Seguin et al., 2005; Weiler et al., 2005). TNFα induces multiple cellular responses, including the decreased expression of both collagen II and aggrecan genes and increased expression of MMP3 and MMP13. In the present study, we revealed that UA protects NP cells from H_2_O_2_-induced senescence and prevents TNFα-induced matrix remodeling. The expression of MMP3, MMP13, and collagen II in the UA + TNFα group was closed to that in the control group. However, it seems to have no influence on aggrecan expression. To investigate further how UA affects the progression of IDD, we studied the influence of UA on NP cell proliferation and H_2_O_2_-induced senescence. In previous studies, H_2_O_2_ was shown to trigger NP cell senescence (Kim et al., 2009; Dimozi et al., 2015; Chen et al., 2016). We observed that UA had no influence on NP cell proliferation and that it could significantly protect NP cells from H_2_O_2_-induced senescence.

**FIGURE 7:**
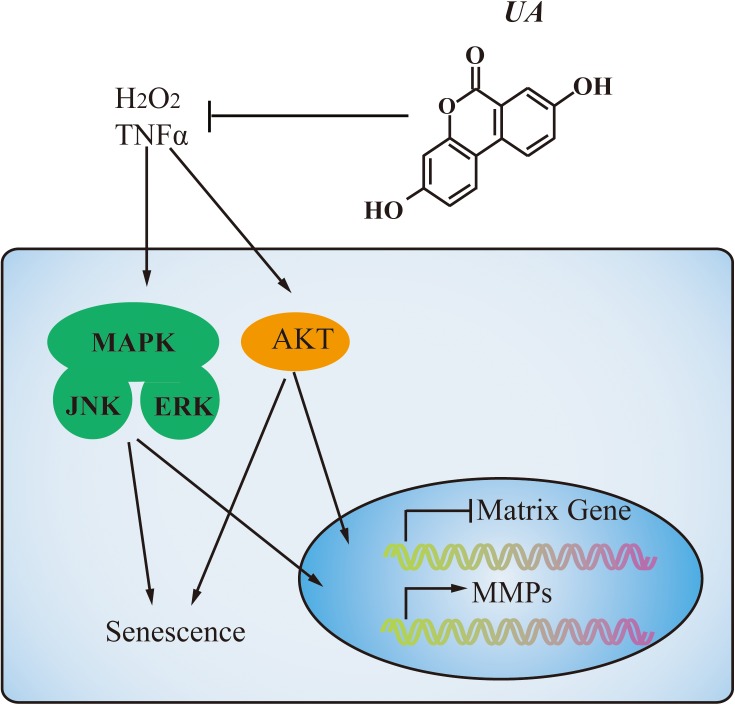
A proposed model of the mechanism. During degeneration, MAPK and PI3K/Akt pathways were activated by TNFα stimulation. UA treatment could decrease MMPs expression and increase matrix gene expression through inhibiting the activation of JNK, ERK, and Akt. Meanwhile, UA could prevent NP cell senescence as well.

Various intracellular signaling pathways are related to IDD. NF-κB and MAPK pathways have been identified as the master regulators of inflammation and catabolism in the process of IDD and PI3K/Akt pathway has been described as a mediator involved in the senescence and apoptosis of NP cells. At the molecular level, UA mainly inhibited the MAPK and PI3K/Akt pathways (**Figure 7**). We showed that UA inhibited TNFα-induced activation of JNK, ERK, and Akt. TNFα could regulate the activation of several cellular signaling pathways in NP cells, including those of the MAPK family (p38, ERK, JNK), NF-κB, PI3K/Akt, and Notch (Risbud et al., 2005; Wang et al., 2013, 2014; Johnson et al., 2015). A previous study showed that UA attenuates LPS-induced neuroinflammation via inhibiting the MAPK, Akt, and NF-κB pathways (Xu et al., 2018), and alleviates myocardial ischemia/reperfusion injury via the PI3K/Akt pathway (Tang et al., 2017). To determine the mechanism by which UA inhibits ECM degradation and senescence, we examined the expression of p65, p38, ERK, JNK, and Akt. The results showed that UA specifically attenuated the TNFα-induced phosphorylation of ERK, JNK, and Akt. However, UA did not influence the phosphorylation of NF-κB p65 and p38 in NP cells. It may be related to the different effects of UA on NF-κB p65 and p38 activity in different cells.

This study has several limitations which have to be pointed out. First, the degenerated NP cells secreted several pro-inflammatory cytokines, including TNFα, IL-1α/β, IL-6, and IL-17, which promote ECM degradation and changes in the cell phenotype, leading to degeneration (Risbud and Shapiro, 2014). We just focused on the role of TNFα in IDD. In addition, different opinions have been expressed concerning the role of TNFα in NP cells (Goupille et al., 2007; Hoyland et al., 2008), and further efforts are needed to solve these problems. Second, we found that UA alleviated IDD in the rat tail using a puncture-induced IDD model. We speculate that UA may reduce or inhibit inflammatory cytokines that are released from NP, similar to the mechanism observed in *in vitro* studies. However, the *in vivo* animal model did not ideally match the *in vitro* model. Third, despite these promising findings, further investigation, such as drug dose and a large animal model, are needed before UA can be considered for clinical use.

## Conclusion

In summary, we demonstrated for the first time that UA has therapeutic effect on IDD. UA could ameliorate TNFα-induced ECM degradation and H_2_O_2_-induced senescence in NP cells via the ERK, JNK, and Akt pathways and alleviate IDD *in vivo*. Administering UA may be an effective treatment for IDD that causes LBP.

## Ethics Statement

The protocol was approved by the Ethics Committee on Animal Experimentation of Tongji Medical College.

## Author Contributions

HL, ZF, and FL were responsible for study design, conducted data analysis, and drafted the manuscript. HL, HK, CS, ZL, LL, JG, YX, and HG conducted the study. HL and HK contributed to data collection. ZF and FL take responsibility for the integrity of the data analysis. All the authors approved the final version of the manuscript.

## Conflict of Interest Statement

The authors declare that the research was conducted in the absence of any commercial or financial relationships that could be construed as a potential conflict of interest.
